# Prenatal polycyclic aromatic hydrocarbon (PAH) exposure in relation to placental corticotropin releasing hormone (pCRH) in the CANDLE pregnancy cohort

**DOI:** 10.3389/fendo.2022.1011689

**Published:** 2022-11-11

**Authors:** Emily S. Barrett, Tomomi Workman, Marnie F. Hazlehurst, Sophie Kauderer, Christine Loftus, Kurunthachalam Kannan, Morgan Robinson, Alicia K. Smith, Roger Smith, Qi Zhao, Kaja Z. LeWinn, Sheela Sathyanarayana, Nicole R. Bush

**Affiliations:** ^1^ Department of Biostatistics and Epidemiology, Rutgers University, Rutgers School of Public Health, Piscataway, NJ, United States; ^2^ Environmental and Occupational Health Sciences Institute, Rutgers University, Piscataway, NJ, United States; ^3^ Department of Environmental and Occupational Health Sciences, University of Washington, Seattle, WA, United States; ^4^ Robert Wood Johnson Medical School, Rutgers University, Piscataway, NJ, United States; ^5^ Department of Pediatrics and Department of Environmental Medicine, New York University School of Medicine, New York, NY, United States; ^6^ Department of Gynecology and Obstetrics, Department of Psychiatry and Behavioral Sciences, Emory University School of Medicine, Atlanta, GA, United States; ^7^ Hunter Medical Research Institute, University of Newcastle, Newcastle, NSW, Australia; ^8^ Department of Preventive Medicine, University of Tennessee Health Science Center, Memphis, TN, United States; ^9^ Department of Psychiatry and Behavioral Sciences, University of California San Francisco, San Francisco, CA, United States; ^10^ Seattle Children’s Research Institute, University of Washington, Seattle, WA, United States; ^11^ Department of Epidemiology, University of Washington, Seattle, WA, United States; ^12^ Department of Pediatrics, University of Washington, Seattle, WA, United States; ^13^ Department of Pediatrics, Division of Developmental Medicine, University of California San Francisco, San Francisco, CA, United States

**Keywords:** polycyclic aromatic hydrocarbons, pregnancy, endocrine disruption, hormones, placenta, corticotropin releasing hormone

## Abstract

Polycyclic aromatic hydrocarbons (PAHs) are ubiquitous endocrine-disrupting combustion by-products that have been linked to preterm birth. One possible mechanism is through disruption of placental corticotropin releasing hormone (pCRH), a key hormone implicated in parturition. As an extension of recent research identifying pCRH as a potential target of endocrine disruption, we examined maternal PAH exposure in relation to pCRH in a large, diverse sample. Participants, drawn from the CANDLE cohort, part of the ECHO-PATHWAYS Consortium, completed study visits at 16-29 weeks (V1) and 22-39 weeks (V2) gestation (n=812). Seven urinary mono-hydroxylated PAH metabolites (OH-PAHs) were measured at V1 and serum pCRH at V1 and V2. Associations between individual log-transformed OH-PAHs (as well as two summed PAH measures) and log(pCRH) concentrations across visits were estimated using mixed effects models. Minimally-adjusted models included gestational age and urinary specific gravity, while fully-adjusted models also included sociodemographic characteristics. We additionally evaluated effect modification by pregnancy complications, fetal sex, and maternal childhood trauma history. We observed associations between 2-OH-Phenanthrene (2-OH-PHEN) and rate of pCRH change that persisted in fully adjusted models (β=0.0009, 0.00006, 0.0017), however, positive associations with other metabolites (most notably 3-OH-Phenanthrene and 1-Hydroxypyrene) were attenuated after adjustment for sociodemographic characteristics. Associations tended to be stronger at V1 compared to V2 and we observed no evidence of effect modification by pregnancy complications, fetal sex, or maternal childhood trauma history. In conclusion, we observed modest evidence of association between OH-PAHs, most notably 2-OH-PHEN, and pCRH in this sample. Additional research using serial measures of PAH exposure is warranted, as is investigation of alternative mechanisms that may link PAHs and timing of birth, such as inflammatory, epigenetic, or oxidative stress pathways.

## Introduction

Polycyclic aromatic hydrocarbons (PAHs) are common environmental pollutants that occur due to incomplete combustion of organic matter (1). PAH exposure occurs through ambient air pollution and tobacco smoke as well as through food sources and occupational hazards, resulting in nearly ubiquitous exposure around the world (2–4). Even among non-smokers, PAH exposure is widespread, with recent research suggesting 96% of non-smoking Americans have detectable levels of one or more urinary PAH metabolites (5). Evidence of PAHs’ carcinogenic, teratogenic, and mutagenic properties (6, 7) has led to their designation as priority pollutants by the United State EPA and the European Commission (8, 9). In addition, research increasingly demonstrates their endocrine disrupting properties, with *in vitro* and animal model evidence indicating impacts on estrogen (10–12), thyroid (13–15), and progesterone pathways (16, 17).

The widespread PAH exposure documented in pregnant people is of particular concern given fetal vulnerability to environmental contaminants, including endocrine disruptors (18). Recent work from our group examined urinary hydroxylated PAH metabolites (OH-PAHs), a common biomarker of PAH exposure, reporting that higher second trimester urinary 2-hydroxynaphthalene (2-OH-NAP) was associated with earlier gestational age at birth and higher 1-hydroxypyrene (1-OH-PYR) with increased odds of spontaneous preterm birth among participants carrying female infants (19). Several studies examining alternative measures of PAH exposure, such PAH-DNA adducts and air monitoring, have similarly reported associations with shorter gestation (20) and increased risk of preterm birth (21, 22). Beyond potential impacts on timing of birth, the detection of PAH biomarkers in cord blood at delivery (23) and spontaneously aborted fetal tissue (24), demonstrates that they can cross the placental barrier to reach the developing fetus, and some evidence suggests associations with adverse downstream child health outcomes, including cognition and behavior (25–27), growth and pubertal development (28, 29), and asthma and allergic outcomes (30–32).

While prenatal exposures to PAHs (and environmental chemicals more generally) are often presumed to impact child development through direct effects on the fetus’ developing tissues, it is also possible that indirect effects may occur through alterations in placental development and physiology, with downstream effects on fetal/child health and development (33). Supporting this premise is prior research showing that PAHs are present in measurable levels in the placenta (23, 34) and in experimental models, PAH exposure reduces trophoblast cell function and viability (35–37).

Given the placenta’s role as a primary endocrine organ during pregnancy, the impact of PAH exposure on placental hormone production merits consideration. Of particular relevance is placental corticotropin releasing hormone (pCRH), which rises exponentially across pregnancy [reviewed in 38]. Mid-late pregnancy pCRH and the rise in pCRH across pregnancy have been linked to preterm birth (39–42), pregnancy complications (43, 44), postpartum depression (45, 46), and offspring development (47, 48). Although the same molecule is also produced by the hypothalamus as part of the hypothalamic-pituitary-adrenal (HPA) axis (49), given that CRH levels are 10,000 times higher in pregnant versus non-pregnant individuals, virtually all detectable CRH in maternal circulation is of placental origin (pCRH). Surprisingly, this important hormone has received little attention in the context of chemical exposures in pregnancy, although several recent studies have shown alterations in pCRH in relation to prenatal maternal exposures to endocrine-disrupting phthalates (50, 51) and per- and poly-fluoroalkyl substances (52).

To our knowledge, to date, only one epidemiological study has examined associations between prenatal OH-PAH exposures and placental hormones, including pCRH (53). In that study of 707 pregnant participants from the PROTECT cohort in Puerto Rico, interquartile (IQR) increases in OH-PAHs in mid-late pregnancy were associated with 14-24% increases in pCRH, with some evidence indicating stronger associations among pregnant people carrying male fetuses.

In light of epidemiological evidence that PAH exposures are associated with shorter gestation as well as results from the PROTECT study indicating direct associations between OH-PAHs and pCRH concentrations, here we extend the current, limited literature on this topic by examining maternal urinary OH-PAHs in relation to pCRH concentrations in mid- and late-pregnancy. We do so within the context of the Conditions Affecting Neurocognitive Development and Learning in Early Childhood (CANDLE) study, a socioeconomically and racially diverse, well-characterized pregnancy cohort located in the Southeastern United States.

## Materials and methods

### Study population and overview

From 2006-2011, pregnant participants were recruited into the CANDLE study through participating prenatal clinics in Shelby County, Tennessee, USA (54). Eligible participants had low medical-risk pregnancies at enrollment, were carrying a singleton fetus, 16-29 weeks pregnant, and 16-40 years old, with plans to deliver at a participating hospital. Low-medical risk was assessed as lacking major medical conditions at the time of consent, including (but not limited to) insulin-dependent diabetes and other endocrine disorders as well as chronic hypertension. Two study visits were conducted, roughly corresponding to mid- (Visit 1 [V1]; 16-29 weeks) and late (Visit 2 [V2]; 22-39 weeks) pregnancy, allowing for flexibility of timing to coordinate with clinical care and minimize participant burden. Prior to any study activities, Institutional Review Board approvals were obtained from the University of Tennessee Health Sciences Center (primary data collection site) and participants signed written, informed consent. The current analysis was facilitated by the NIH’s Environmental Influences of Child Health Outcomes (ECHO) program and in particular, the ECHO-PATHWAYS Consortium.

Inclusion in the current analysis was determined based on having data on urinary OH-PAH concentrations (measured at V1) as well as plasma pCRH concentrations (measured at V1 and V2). Given the strong impact of active smoking on PAH exposures as well as pregnancy physiology, in our main analyses, we excluded women who self-reported smoking during pregnancy and/or had urinary cotinine levels exceeding 200 ng/mL (55).

### Exposure assessment: Maternal urinary PAH metabolites

At V1 in mid-pregnancy, participants provided a spot urine sample. Specific gravity (SG) was measured with a handheld refractometer after which samples were aliquoted and frozen at -80°C until shipment on dry ice to the Wadsworth Laboratory, New York State Department of Health, Albany, New York. Using methods previously described elsewhere (56), twelve OH-PAH metabolites were measured including: two metabolites of naphthalene (1-OH-NAP, 2-OH-NAP), four metabolites of phenanthrene (2-hydroxyphenanthrene [2-OH-PHEN], 3-hydroxyphenanthrene [3-OH-PHEN], 4-hydroxyphenanthrene [4-phen], combined 1/9-hydroxyphenanthrene [1/9-OH-PHEN]), combined 2/3/9-hydroxyfluorene (2/3/9-OH-FLUO), 1-OH-PYR, 3-hydroxybenzo[c]phenanthrene (3-BCP), two metabolites of hydroxychrysene (1-hydroxychrysene [1-OH-CHRY], 6-hydroxychrysene [6-OH-CHRY]), and 1-hydroxybenz[a]anthracene (1-OH-BAA). Briefly, 10 ng of isotopically-labeled internal standard mixture was added to 500 µL urine samples, and then combined with 1 mL of 0.5 M ammonium acetate buffer containing 200 units/mL of β-glucuronidase/sulfatase enzyme (MP Biomedicals, LLC, Solon, OH, USA). Following an overnight incubation, the sample mixtures were diluted with 2 mL of HPLC-grade water and extracted with a 7 mL pentane:toluene solution for one hour. Samples were centrifuged for 20 minutes at 3600 x g and the resulting supernatant was transferred for instrumental analysis. A Waters Acquity I-Class UPLC system (Waters; Milford, MA, USA) connected with an Eclipse Plus C18 RRHD column (100 mm × 2.1 mm, 1.8 μm, Agilent; Santa Clara, CA, USA) was used for chromatographic separation of the OH-PAH metabolites followed by quantification using an ABSCIEX 5500 triple quadrupole mass spectrometer (Applied Biosystems; Foster City, CA, USA). Two Standard Reference Materials (SRM 3672, SRM 3673) were used as quality assurance protocols and analyte recovery in the SRMs was 79-109%. To ensure instrument stability, during the sample run, calibration standards were periodically injected. The limits of detection varied by metabolite ranging from 0.02-0.12 ng/mL We limited the current analyses to seven PAH metabolites above the LOD in >60% of samples, specifically: 1-OH-NAP, 2-OH-NAP, 2-OH-PHEN, 3-OH-PHEN, 1/9-OH-PHEN, 2/3/9-OH-FLUO, and 1-OH-PYR. For these metabolites, values below the LOD were replaced with LOD/√2. We additionally calculated ∑NAP as the sum of 1-OH-NAP and 2-OH-NAP, and ∑PHEN as the sum of 2-OH-PHEN, 3-OH-PHEN, 4-OH-PHEN, and 1/9-OH-PHEN.

### Outcome assessment: pCRH

At V1 and V2, participants provided blood samples in EDTA plasma separator tubes. Blood was processed and frozen at -80°CC until it was shipped on dry ice to University of Newcastle, Australia for pCRH analysis. pCRH was measured in pg/mL using radioimmunoassay according to previously published protocols (57). The inter- and intra-assay coefficients of variation were 8.7% and 7.3% respectively. pCRH concentrations were non-normally distributed and were therefore log-transformed for subsequent analysis.

### Covariates

Data on key covariates, selected *a priori* based on the literature, were collected from questionnaires administered during pregnancy as well as from clinical chart reviews. Participants reported on age, highest level of educational attainment (categorized for this analysis as less than high school, high school/GED/technical school, or college or higher), parity (parous/nulliparous), and pre-pregnancy weight and height (used to calculate body mass index in kg/m^2^). Participants also reported race and ethnicity, which were included here (categorized as non-Hispanic White, non-Hispanic Black, Hispanic, or Other) as proxies for chronic exposure to discrimination and systemic racism, which may contribute to variation in exposures as well as alter endocrine activity in pregnancy. Relevant to our focus on pCRH, participants additionally reported on history of childhood exposures to traumatic stressors *via* three items from the Traumatic Life Events Questionnaire (TLEQ) specifically querying history of physical abuse and family violence before age 18 and sexual abuse prior to age 13. These items were used to construct a counts (discrete) variable for total number of types of childhood traumatic exposures, ranging from 0-3 (58, 59). Gestational age at sample collection was determined based on the medical record. In general, clinical determination was based on date of last menstrual period and confirmed with ultrasound dating, with the latter being prioritized in cases of discrepancies. Maternal gestational diabetes and hypertension were self-reported shortly after delivery, then confirmed by medical record abstraction and are included in the current analysis based on prior work indicating associations with pCRH in this cohort (51, 60). Finally, although participants who reported actively smoking or had cotinine levels >200 ng/mL were excluded from our primary analyses, exposure to environmental tobacco smoke was assessed by measurement of cotinine in spot urine samples collected at V1 (considered continuously in models).

### Statistical analysis

Participants who: (1) had OH-PAH metabolite data; (2) had pCRH data at one or more timepoint; and (3) did not report smoking during pregnancy (and had cotinine ≤200 ng/mL), were eligible for inclusion in our primary analyses. We calculated descriptive statistics to characterize the sample including geometric mean and SD, median, min, max, quartiles, %<LOD, percentages, and frequencies) followed by bivariate analyses to examine relationships between the variables of interest, including the exposures and outcomes. We used Spearman correlations to examine associations among the OH-PAHs.

We fitted a set of individual linear regression models for each of the seven OH-PAHs included in the current analysis as well as two summed PAH variables (ΣNAP and ΣPHEN). For each OH-PAH, we examined the rate of change in pCRH between V1 and V2 using a mixed effects model with gestational day at pCRH sampling as a continuous variable and random intercepts for subjects. For each metabolite, we fit three staged models (minimally adjusted, fully adjusted, and extended), with covariates determined *a priori* based on the literature, particularly our recent publications on pCRH in this cohort (51, 60). Minimally adjusted models considered only those covariates most relevant to the biomarker measures themselves, namely specific gravity (as a measure of urine dilution) and gestational age at blood collection (relevant given the strong temporal changes in pCRH across pregnancy). Our primary, fully adjusted models additionally included maternal and pregnancy-related covariates associated with pCRH concentrations (and in some cases, with OH-PAH concentrations) including maternal age, race/ethnicity, education, pre-pregnancy BMI, cotinine, parity, childhood traumatic events, and fetal sex. In extended models, we additionally considered gestational diabetes and gestational hypertension as they have been confounders of pCRH in prior work, but could arguably be on the pathway between exposure and outcome (61, 62). Results are reported for the interaction between each of the OH-PAHs and time in days. Additionally, based on our prior work on pCRH in this cohort, we evaluated effect modification by the binary variables fetal sex, maternal childhood traumatic events (any/none), gestational hypertension, and gestational diabetes (51). To do so we refitted extended models adding a multiplicative interaction term (e.g. OH-PAH*fetal sex) with the p-value for the interaction term used to determine statistical significant differences by group (at alpha level=0.05).

Secondarily, we fit linear regression models examining pCRH at the two outcome timepoints (V1 and V2) separately. We additionally conducted a series of sensitivity analyses to evaluate the robusticity of our results. First, we refitted minimally adjusted, fully adjusted, and extended models using PAH levels pre-adjusted for specific gravity before model fitting (rather than including specific gravity as a covariate in the primary analysis) using the formula:


$$P_{c}=P*\left[\frac{SG_{median}-1}{SG-1}\right]$$

In this formula, P represents measured urinary OH-PAH concentration, SG represents individual participants’ specific gravity, and *SG*_*median*_ is the median SG for the batch (63). We additionally refitted models: (1) including smokers and women with urinary cotinine >200ng/mL; (2) omitting preterm (<37 weeks) and post-term (>42 weeks) births; (3) omitting women with a history of prior preterm birth; and (4) without adjustment for childhood traumatic events. Finally, to avoid concerns around overlap in timing of V1 and V2, we refit models that more strictly adhered to trimester definitions (e.g. only including V1 occurring prior to 27 weeks and V2 occurring after 27 weeks gestation). All analyses were conducted using R 4.1.3 (R Foundation for Statistical Computing, Vienna, Austria).

## Results

In total, 812 CANDLE participants were included in our main analyses (**Table 1**). Participants were on average 26.8 ± 5.6 years old at enrollment with a pre-pregnancy BMI of 28.1 ± 7.8 kg/m^2^. Most participants were non-Hispanic Black (62.0%) or non-Hispanic White (32.0%), with few identifying as Hispanic (1.0%) or another race (5.0%). The majority of participants had a high school/GED/or technical school-level education (55.0%), with an additional 37.0% completing college or higher. In total, 41.0% of participants were nulliparous and just over half (52.0%) were carrying female fetuses. A small percentage of participants developed gestational diabetes (6.0%) or gestational hypertension (10.0%) during the index pregnancy.

**Table 1:** Characteristics of CANDLE mother-child dyads (n=812).

Characteristics (continuous)	Mean ± SD
Maternal age (years)	26.8 ± 5.6
Pre-pregnancy BMI (kg/m^2^)	28.1 ± 7.8
Gestational age at Visit 1 (V1; weeks)	22.9 ± 3.1
Gestational age at Visit 2 (V2; weeks)	31.8 ± 1.6
Change in gestational age (V2-V1; weeks)	8.9 ± 3.1
Maternal Childhood Traumatic Life Events	0.5 ± 0.8
Maternal Adult Traumatic Life Events	3.2 ± 2.3
pCRH at V1 (pg/mL)	57.1 ± 77.9
pCRH at V2 (pg/mL)	360.3 ± 431.7
Urinary cotinine (ng/mL)	2.5 ± 7.5
**Characteristics (categorical)**	**N (%)**
Maternal race/ethnicity Non-Hispanic Black Non-Hispanic White Hispanic Other	503 (62.0) 256 (32.0) 12 (1.0) 41 (5.0)
Highest level of maternal education<High school High school/GED/Technical School College or higher	65 (8.0) 444 (55.0) 302 (37.0)
Nulliparous	329 (41.0)
Gestational diabetes	47 (6.0%)
Gestational hypertension	82 (10.0%)
Fetal sex-female	420 (52.0%)

Of the OH-PAHs measured, 1-OH-NAP, 2-OH-NAP, 3-OH-PHEN, and 2/3/9-OH-FLUO were found in >96% of participants, with average 2-OH-NAP concentrations being highest (geometric mean 5.37 ± 2.41 ng/mL; **Table 2**). Correlations between the OH-PAH metabolites tended to be moderate to high, ranging from a low of r=0.22 for 1/9-OH-PHEN and 2-OH-NAP to a high of 0.85 for 2-OH-PHEN and 3-OH-PHEN (**Supplementary Table S1**). Overall, PAH metabolites tended to be higher among younger participants, less educated participants, participants with higher BMI, and non-Hispanic Black participants (compared to non-Hispanic White; **Supplementary Table S2**).

**Table 2:** Maternal urinary PAH metabolites at Visit 1 in ng/mL (n=812).

OH-PAH metabolite	LOD	%<LOD^1^	Min.	5^th^ %	25^th^ %	50^th^%	75^th^%	95th%	Max.	Geo. Mean	Geo. SD
1-OH-Naphthalene (1-OH-NAP)	0.02	0	0.08	0.30	0.61	1.15	2.32	11.82	367.78	1.34	3.30
2-OH-Naphthalene (2-OH-NAP)	0.025	0.25	0.01	1.47	3.09	5.41	8.77	20.63	151.99	5.37	2.41
2-OH-Phenanthrene (2-OH-PHEN)	0.03	14.29	0.01	0.04	0.07	0.10	0.15	0.32	4.13	0.10	1.94
3-OH-Phenanthrene (3-OH-PHEN)	0.03	0	0.01	0.04	0.07	0.10	0.15	0.30	2.99	0.10	1.90
4-OH-Phenanthrene (4-OH-PHEN)	0.03	58.13	0.01	0.02	0.03	0.04	0.06	0.14	0.91	0.04	1.95
1/9-OH-Phenanthrene (1/9-OH-PHEN)	0.08	16.50	0.01	0.02	0.20	0.37	0.62	1.37	13.07	0.31	3.15
2/3/9-OH-Fluorene (2/3/9-OH-FLUO)	0.12	3.94	0.04	0.35	0.66	0.97	1.54	3.44	32.48	1.02	2.05
1-OH-Pyrene (1-OH-PYR)	0.03	11.82	0.02	0.05	0.09	0.15	0.25	0.55	3.38	0.15	2.15
1-hydroxybenz[a]anthracene (1-OH-BAA)	0.03	100	0.01	0.02	0.02	0.02	0.04	0.08	0.42	0.03	1.74
3-hydroxybenzo[c]phenanthrene (3-BCP)	0.025	100	0.01	0.01	0.02	0.02	0.03	0.07	0.35	0.02	1.74
1-hydroxychrysene (1-OH-CHRY)	0.02	100	0.01	0.01	0.01	0.02	0.02	0.06	0.28	0.02	1.74
6-hydroxychrysene (6-OH-CHRY)	0.025	100	0.01	0.01	0.02	0.02	0.03	0.07	0.35	0.02	1.74

OH-PAH, monohydroxy-polycyclic aromatic hydrocarbon; LOD, limit of detection; Min., Minimum; Max, Maximum; Geo. Mean, geometric mean; Geo SD, geometric standard deviation.

1 Only metabolites with<40% of samples below the LOD were included in subsequent analyses.

2 Individual values below the LOD were imputed as LOD/(sqrt2).

In mixed effect models integrating both outcome timepoints, all interaction terms for OH-PAHs and gestational days (time) indicated positive associations with the rate of the pCRH rise (**Table 3**). In minimally adjusted models, the strongest associations with the rate of the pCRH rise were observed for 2-OH-PHEN (β=0.0010; 95% CI:0.0002, 0.0018), 3-OH-PHEN (β=0.0008; 95% CI: -0.000002, 0.0016); and 1-OH-PYR (0.0007; 95% CI: -0.00003, 0.0013). In fully adjusted models, after adjustment for sociodemographic covariates, associations with the rate of the pCRH rise were moderately attenuated for all three metabolites: 2-OH-PHEN (β=0.0009; 95% CI:0.00006, 0.0017), 3-OH-PHEN (β=0.0007; 95% CI: -0.0002, 0.0015); and 1-OH-PYR (β=0.0005; 95% CI: -0.0002, 0.0010). Results of the extended models were similar to those of the fully adjusted models, with all confidence intervals including the null, with the exception of 2-OH-PHEN (β=0.0008; 95% CI:0.00002, 0.0016). In analyses evaluating effect modification by fetal sex, maternal exposure to childhood trauma, gestational diabetes, and gestational hypertension, little evidence of effect modification was observed (**Supplementary Table S3**).

**Table 3:** Mixed effect models examining log-transformed PAH metabolite concentrations in relation to change in pCRH concentrations across mid-late pregnancy.

OH-PAH metabolite^1,2^	Minimally adjusted model^3^β (95% CI); p-value	Fully adjusted model^4^β (95% CI); p-value	Extended model^5^β (95% CI); p-value
	N=812	N=797	N=792
1-OH-NAP	0.0002 (-0.0002, 0.0006); 0.34	0.0001 (-0.0003, 0.0006); 0.49	0.0002 (-0.0002, 0.0006); 0.27
2-OH-NAP	0.0003 (-0.0002, 0.0009); 0.25	0.0003 (-0.0003, 0.0009); 0.33	0.0003 (-0.0003, 0.0009); 0.31
2-OH-PHEN	0.0010 (0.0002, 0.0018); 0.01	0.0009 (0.00006, 0.0017); 0.03	0.0008 (0.00002, 0.0016); 0.05
3-OH-PHEN	0.0008 (-0.00002, 0.0016); 0.06	0.0007 (-0.0002, 0.0015); 0.12	0.0007 (-0.00009, 0.0015); 0.08
1/9-OH-PHEN	0.0002 (-0.0003, 0.0006); 0.47	0.0009 (-0.0002, 0.0006); 0.39	0.0003 (-0.0001, 0.0007); 0.19
2/3/9-OH-FLUO	0.0003 (-0.0004, 0.0010); 0.47	0.00009 (-0.0006, 0.0008); 0.81	0.0001 (-0.0006, 0.0008); 0.79
1-OH-PYR	0.0007 (-0.000003, 0.0013); 0.05	0.0005 (-0.0002, 0.0012); 0.15	0.0006 (-0.00005, 0.0013); 0.07
∑NAP	0.0005 (-0.00012, 0.0010); 0.12	0.0004 (-0.0002, 0.0010); 0.18	0.0005 (-0.0001, 0.0010); 0.12
∑PHEN	0.0004 (-0.0003, 0.0011); 0.31	0.0003 (-0.0004, 0.0011); 0.36	0.0005 (-0.0003, 0.0012); 0.20

1 1-OH-NAP= 1-OH-Naphthalene; 2-OH-NAP=2-OH-Naphthalene; 2-OH-PHEN= 2-OH-Phenanthrene; 3-OH-PHEN= 3-OH-Phenanthrene; 1/9-OH-PHEN=combined 1/9-OH-Phenanthrene; 2/3/9-OH-FLUO= combined 2/3/9-OH-Fluorene; 1-OH-PYR= 1-OH-Pyrene; ∑NAP=sum of 1-OH-NAP and 2-OH-NAP; ∑PHEN=sum of 2-OH-PHEN, 3-OH-PHEN, 4-OH-PHEN, and 1/9-OH-PHEN.

2 Coefficients, 95% confidence intervals, and the p-values for the interaction between each of the log-transformed OH-PAHs and time (repeated measures of gestational age at blood collection) are reported.

3 Minimally adjusted models include gestational age at blood collection, specific gravity.

4 Fully adjusted models include gestational age at blood collection, specific gravity, maternal age, race/ethnicity, education, pre-pregnancy BMI, cotinine, fetal sex, parity, and childhood traumatic events as well as interaction terms for each of the covariates * gestational age at blood collection.

5 Extended models include gestational age at blood collection, specific gravity, maternal age, race/ethnicity, education, pre-pregnancy BMI, cotinine, fetal sex, parity, childhood traumatic events, gestational diabetes, and gestational hypertension as well as interaction terms for each of the covariates * gestational age at blood collection.

In secondary models examining the association between OH-PAH concentrations and pCRH at individual timepoints (**Supplementary Table S4**), we similarly observed associations in minimally adjusted models that were attenuated after adjustment for covariates. In minimally adjusted models, examining pCRH at V1, we observed significant associations with 3-OH-PHEN (β=-0.09, 95%CI: -0.16, -0.01), 2/3/9-OH-FLUO (β=-0.07, 95% CI: -0.13, -0.001), 1-OH-PYR (β=-0.10, 95%CI: -0.16, -0.03), and ∑NAP (β=-0.06, 95% CI: -0.12, -0.01), with a trend towards association observed for 2-OH-PHEN (β=-0.07, 95%CI: -0.14, 0.01). Most associations between OH-PAHs and V2 pCRH were inverse, but non-significant. Only 1-OH-PYR was significantly associated with lower V2 pCRH (β=-0.08, 95% CI: -0.15, -0.004). However, in fully adjusted models including sociodemographic and lifestyle-related covariates, associations were generally in the positive direction, though estimates were small in magnitude and 95% confidence intervals included the null across all OH-PAHs and all outcome measures (**Supplementary Table S4**). In extended models also including pregnancy complications, results were nearly identical to the fully adjusted models and again, null for all exposure-outcome associations.

In sensitivity analyses using specific gravity-adjusted OH-PAH values rather than including specific gravity as a covariate, results were unchanged (not shown). We additionally refit models to also including smokers and participants with urinary cotinine >200 ng/mL (an additional 119 participants), observing that overall, associations were similar, but modestly strengthened (**Supplementary Table S5**), with significant associations observed for 2-OH-PHEN and 3-OH-PHEN in the fully adjusted models. Results were similarly robust to the exclusion of preterm (<37 weeks) and post-term (>42 weeks) births (68 participants excluded), with positive associations and/or trends observed for 2-OH-PHEN, 3-OH-PHEN, and 1-OH-PYR in fully adjusted models (**Supplementary Table S6**). Results were virtually unchanged when we restricted analyses to participants with no history of prior preterm birth (60 participants excluded), although associations with 1-OH-PYR were stronger and reached statistical significance (β=0.008; 95% CI: 0.001, 0.0015; **Supplementary Table S7**). Results were nearly identical to our main models in sensitivity analyses excluding maternal childhood traumatic events as a covariate (**Supplementary Table S8**). Finally, patterns of association were robust to stricter adherence to visit timing by trimester (i.e. analyses restricted to participants with V1 occurring at or prior to 27 weeks and V2 occurring after 27 weeks gestation; **Supplementary Table S9**), although estimates were modestly attenuated, likely due to the smaller sample size (n=713).

## Discussion

In this analysis, we examined associations between OH-PAHs and pCRH in a large, socioeconomically and racially diverse pregnancy cohort, observing that higher maternal 2-OH-PHEN concentrations (and to a lesser extent, 3-OH-PHEN and 1-OH-PYR) were associated with a more rapid rate of increase in pCRH in mid-late pregnancy, after adjustment for covariates. In models examining pCRH at individual timepoints, associations between OH-PAHs and pCRH in mid-pregnancy were attenuated after adjustment for sociodemographic and lifestyle factors, and no associations with late pregnancy pCRH were observed. We additionally observed no evidence of effect modification by fetal sex, childhood traumatic history, or pregnancy complications. Overall, our results suggest that maternal PAH exposure, particularly to 2-OH-PHEN, 3-OH-PHEN, and 1-OH-PYR, may amplify pCRH production in mid-late pregnancy, extending the limited prior literature on PAHs and pCRH.

In the prior study on this topic in the Puerto Rico-based PROTECT cohort (n=659), across nearly all metabolites, participants with higher PAH exposures had considerably higher pCRH concentrations, echoing the positive associations observed in our mixed effect models (50). In PROTECT, comparing women in the highest quartile of PAH exposure relative to the lowest, pCRH concentrations were higher in relation to 1-OH-NAP (14.0%, 95% CI: 4.06%, 24.9%), 1-OH-PYR (9.21%; 95% CI: -1.93%, 21.6%), 2-OH-FLU (15.30%; 95% CI: 4.54%, 27.10%), 1-OH-PHE (24.3%; 95% CI: 13.0%, 36.7%), and sum2,3-OH-PHE (18.1%, 95% CI: 7.00%, 30.4%). We similarly observed positive associations in relation to 1-OH-PYR as well as with 2-OH-PHEN and 3-OH-PHEN (considered individually in our study, but summed in PROTECT). However, in contrast to that study, we did not observe associations between pCRH and 1-OH-NAP nor the 2/3/9-hydroxyfluorene metabolites. In PROTECT, associations tended to be stronger in early in pregnancy (16-20 weeks) compared to the later visits (20-24 and 24-28 weeks); while we also observed stronger associations with pCRH in mid- (versus late) pregnancy, in models examining individual outcome timepoints, results were attenuated and confidence intervals included the null after adjustment for sociodemographic factors.

Despite the different geographic study locations, with likely differences in patterns of exposure to air pollutants as well as dietary sources of PAHs, concentrations of the four OH-PAHs measured in common across both studies (1-OH-NAP, 2-OH-NAP,1-OH-PYR, 4-OH-PHEN) were similar and, in some cases, slightly higher in CANDLE as compared to PROTECT. Across the two cohorts, pCRH concentrations were similar at the earlier study visit, with CANDLE median levels of 57.1 pg/mL at 16-29 weeks gestation and PROTECT median levels of 56.3 pg/mL at 16-20 weeks gestation. However, at the later study visit, CANDLE median pCRH levels rose to 360.3 pg/mL, whereas PROTECT levels stayed flat at 54.9 pg/mL. While some of this difference may be attributable to differences in the timing of the later visit (22-39 weeks in CANDLE vs 24-28 weeks in PROTECT), a more likely explanation is the use of enzyme-linked immunosorbent assay (ELISA) to perform the assays in PROTECT as compared to RIA in CANDLE. A recent study of 169 women with a prior preterm birth compared pCRH measured both by RIA and ELISA, showed that pCRH concentrations measured by ELISA were stable across gestation, whereas the same samples assayed using RIA showed a steep rise in late pregnancy (64). The flat pCRH levels across pregnancy as measured by ELISA are likely due to binding protein (CRH-BP) interference, which could bias results. While it is possible to perform ELISA with an extraction step that reduces this interference, more commonly that step is not included. Of note, in the comparison study, pCRH levels measured by RIA (but not ELISA) were associated with preterm birth, providing further evidence of pCRH as a “placental clock”.

The positive associations observed between OH-PAH concentrations and pCRH in the PROTECT study were hypothesized to be evidence of a potential mechanism by which PAHs may contribute to preterm birth (53). At present, evidence linking PAHs to preterm birth has been somewhat inconsistent, possibly due to methodological differences regarding study design, inclusion of smokers, preterm birth subtypes, adjustment for confounders, and varied approaches to exposure ascertainment (19–22, 65, 66). Within studies, results can be similarly inconsistent; for instance, in a Fresno, California study, participants in the highest quartile of PAH exposure during the last six weeks of pregnancy had 2.74 times the odds of delivering extremely preterm (20-27 weeks gestation) compared to the lowest quartile (21). However, the association did not hold for later preterm deliveries and, in some models examining other exposure windows, associations appeared to be protective. Most recently, we analyzed harmonized data from CANDLE and two additional cohorts, observing that 10-fold higher second trimester 2-OH-NAP concentrations were associated with 1.6 day shorter gestation, with some evidence of greater vulnerability to spontaneous preterm birth among female fetuses (19). In the current analysis associations between 2-OH-NAP and pCRH were weak and included the null. Conversely, OH-PAH metabolites associated in this analysis with higher rate of pCRH change across pregnancy (such as 2-OH-PHEN) showed little association with preterm birth in that prior analysis. Overall, these results suggest that if there are associations between OH-PAHs and preterm birth and/or shorter gestation, alternative mechanisms may warrant consideration, including induction of oxidative stress (65), changes in DNA methylation (20, 67), and disruption of other placental hormones, such as estriol (53).

A notable strength of this study is the socioeconomic and racial diversity of the sample, which included participants across a broad range of SES and was comprised of over 60% non-Hispanic Black women, a group often underrepresented in pregnancy cohort studies. Our use of a urinary biomarker of PAH exposure is also a strength of the current analysis. The urinary biomarker reflects exposure through multiple sources, including diet, which may account for over 90% of PAH exposure in non-smokers (68, 69). We used gold standard RIA for pCRH analysis of maternal plasma, which is considered superior to pCRH assays conducted using other analytic approaches or sample types (64, 70). Given the well-characterized nature of the CANDLE cohort, moreover, we were able to assess a number of confounders and potential effect modifiers that could influence our analyses; indeed, the significant associations observed in some minimally adjusted models were attenuated after adjustment for factors related to the social determinants of health, such as education, race, and childhood trauma. We observed inequalities in exposure such that OH-PAH concentrations were notably higher among Black and Hispanic participants (compared to non-Hispanic White), participants with lower levels of educational attainment, and overweight or obese participants. The higher OH-PAH concentrations observed in some sub-populations are likely the product of systemic racism and discrimination that leads to greater environmental pollution in marginalized communities. Our results were robust to a number of sensitivity analyses, including restricting the sample to term births, suggesting that associations were not driven by women with prior preterm birth history, who may have differing pCRH profiles.

We note several limitations of the current work. First, OH-PAH metabolites have a half-life of approximately 2-6 hours in the body (71) and in other studies, intra-class correlations characterizing OH-PAH concentrations across pregnancy have been in the low to moderate range (72). In this study, OH-PAHs were measured at a single timepoint in mid-pregnancy raising the possibility of exposure misclassification. Future work examining repeated OH-PAH exposures across pregnancy in relation to placental hormone production would be beneficial. Finally, although the diversity of the sample and its representativeness of Shelby County, Tennessee is a strength and findings are likely generalizable to other, understudied communities in the U.S. South, we cannot assume generalizability to the U.S. as a whole and certain sub-groups, like Latina and Asian women, are under-represented in our analysis. Additionally, by design, CANDLE participants were of relatively low medical risk at the time of recruitment and although some women ultimately developed pregnancy complications, by excluding higher risk pregnancies and women with major medical conditions (e.g. chronic hypertension requiring therapy, endocrine disease, and insulin-dependent diabetes) at enrollment, we may have reduced our ability to detect associations.

In conclusion, in this large pregnancy cohort, we observed some, limited evidence that mid-pregnancy OH-PAH metabolites, particularly 2-OH-PHEN, 3-OH-PHEN, and 1-OH-PYR, are associated with a more rapid increase in production of the placental hormone pCRH in mid to late pregnancy. Future research on disruption of placental hormone activity should examine OH-PAHs repeatedly across pregnancy and additional work is needed to identify alternative biological mechanisms that may link PAHs to preterm birth.

## Data availability statement

The data analyzed in this study is subject to the following licenses/restrictions: The datasets analyzed for this study are not publicly available but de-identified data may be available on request, subject to approval by the internal review board and under a formal data use agreement. Contact the corresponding author for more information. Requests to access these datasets should be directed to emily.barrett@eohsi.rutgers.edu.

## Ethics statement

The studies involving human participants were reviewed and approved by University of Tennessee Health Sciences Center Institutional Review Board. The patients/participants provided their written informed consent to participate in this study.

## Author contributions

EB led the analysis concept and manuscript writing and revision. TW, MH, SK, and CL led data analysis and interpretation and edited the manuscript. KK and MR conducted the chemical analyses, drafted manuscript sections, and edited the manuscript. RS conducted pCRH analyses and edited the manuscript. AS and QZ contributed to study design and implementation and edited the manuscript. KL, SS, and NB obtained funding, led study design, and edited the manuscript.

## Funding

The ECHO PATHWAYS Consortium is funded by NIH UG3/UH3OD023271. The CANDLE study is funded by the Urban Child Institute as well as CIHR award number MWG-146331. Additional support for this analysis was provided by NIH P30ES005022 and UW NIEHS sponsored Biostatistics, Epidemiologic and Bioinformatic Training in Environmental Health (BEBTEH) Training Grant: NIEHS T32ES015459. This manuscript has been reviewed by PATHWAYS for scientific content and consistency of data interpretation with previous PATHWAYS publications. The content is solely the responsibility of the authors and does not necessarily represent the official views of the National Institutes of Health.

## Acknowledgments

We thank the CANDLE participants as well as the staff of CANDLE and ECHO PATHWAYS.

## Conflict of interest

The authors declare that the research was conducted in the absence of any commercial or financial relationships that could be construed as a potential conflict of interest.

## Publisher’s note

All claims expressed in this article are solely those of the authors and do not necessarily represent those of their affiliated organizations, or those of the publisher, the editors and the reviewers. Any product that may be evaluated in this article, or claim that may be made by its manufacturer, is not guaranteed or endorsed by the publisher.
