# Supplementary material for: Prenatal polycyclic aromatic hydrocarbon (PAH) exposure in relation to placental corticotropin releasing hormone (pCRH) in the CANDLE pregnancy cohort
Source: Front Endocrinol (Lausanne). 2022 Nov 11;13:1011689. doi: 10.3389/fendo.2022.1011689 (PMC9691680; doi:10.3389/fendo.2022.1011689)
Supplement: Supplementary file 1 [file DataSheet_1.docx]

Supplementary Material

# Supplementary Tables

**Supplementary Table 1. Spearman correlations between urinary OH-PAH metabolites in the CANDLE cohort (n=812)^1^.**

|  | 1-OH-NAP | 2-OH-NAP | 2-OH-PHEN | 3-OH-PHEN | 1/9-OH-PHEN | 2/3/9/-OH-FLUO | 1-OH-PYR |
| --- | --- | --- | --- | --- | --- | --- | --- |
| 1-OH-NAP | 1.000 | 0.349 | 0.244 | 0.335 | 0.277 | 0.259 | 0.209 |
| 2-OH-NAP |  | 1.000 | 0.350 | 0.363 | 0.219 | 0.467 | 0.286 |
| 2-OH-PHEN |  |  | 1.000 | 0.848 | 0.512 | 0.628 | 0.532 |
| 3-OH-PHEN |  |  |  | 1.000 | 0.519 | 0.668 | 0.565 |
| 1/9-OH-PHEN |  |  |  |  | 1.000 | 0.446 | 0.432 |
| 2/3/9-OH-FLUO |  |  |  |  |  | 1.000 | 0.499 |
| 1-OH-PYR |  |  |  |  |  |  | 1.000 |

1 Abbreviations: 1-OH-NAP= 1-OH-Naphthalene; 2-OH-NAP=2-OH-Naphthalene; 2-OH-PHEN= 2-OH-Phenanthrene; 3-OH-PHEN= 3-OH-Phenanthrene; 1/9-OH-PHEN= combined 1/9-OH-Phenanthrene; 2/3/9-OH-FLUO= combined 2/3/9-OH-Fluorene; 1-OH-PYR= 1-OH-Pyrene

| Supplementary Table 2. Geometric means and standard deviations of specific-gravity adjusted OH-PAH concentrations by covariates. | | | | | | | | | | | | | | | |  |  |
| --- | --- | --- | --- | --- | --- | --- | --- | --- | --- | --- | --- | --- | --- | --- | --- | --- | --- |
|  | **1-OH-NAP** | | **2-OH-NAP** | | **2-OH-PHEN** | | **3-OH-PHEN** | | **1/9-OH-PHEN** | | **2/3/9-OH-FLUO** | | **1-OH-PYR** | |  |  |  |
|  | *Geo. Mean* | *Geo. SD* | *Geo. Mean* | *Geo. SD* | *Geo. Mean* | *Geo. SD* | *Geo. Mean* | *Geo. SD* | *Geo. Mean* | *Geo. SD* | *Geo. Mean* | *Geo. SD* | *Geo. Mean* | *Geo. SD* |  |  |  |
| OVERALL | 1.3424 | 3.2990 | 5.3741 | 2.4115 | 0.1035 | 1.9434 | 0.1049 | 1.9034 | 0.3079 | 3.1487 | 1.0172 | 2.0478 | 0.1545 | 2.1450 |  |  |  |
| Maternal age (years) |  |  |  |  |  |  |  |  |  |  |  |  |  |  |  |  |  |
| 16-20 | 1.6546 | 4.6880 | 6.5682 | 2.8088 | 0.1015 | 2.0132 | 0.1196 | 1.9885 | 0.3208 | 3.3050 | 1.1081 | 2.0117 | 0.1949 | 2.0500 |  |  |  |
| 20-25 | 1.3593 | 2.9267 | 5.9485 | 2.1436 | 0.1061 | 1.8892 | 0.1075 | 1.8579 | 0.2943 | 3.0775 | 1.0959 | 2.0151 | 0.1749 | 2.0980 |  |  |  |
| 25-30 | 1.4480 | 3.2631 | 5.0988 | 2.5296 | 0.1035 | 2.0631 | 0.1019 | 1.9489 | 0.3096 | 3.2123 | 1.0715 | 1.9921 | 0.1590 | 2.2093 |  |  |  |
| 30-35 | 1.1414 | 2.8487 | 4.9003 | 2.3235 | 0.1018 | 1.7962 | 0.1006 | 1.8144 | 0.2914 | 3.1200 | 0.9047 | 1.9808 | 0.1268 | 1.9103 |  |  |  |
| 35-40 | 1.1969 | 3.9090 | 4.7648 | 2.4052 | 0.1028 | 2.0138 | 0.1023 | 1.9871 | 0.3731 | 3.0559 | 0.8534 | 2.4052 | 0.1249 | 2.4337 |  |  |  |
| Maternal race/ethnicity |  |  |  |  |  |  |  |  |  |  |  |  |  |  |  |  |  |
| Non-Hispanic Black | 1.5314 | 3.7004 | 6.6160 | 2.3214 | 0.1120 | 1.9947 | 0.1173 | 1.9180 | 0.3139 | 3.3011 | 1.2045 | 1.9616 | 0.1787 | 2.1048 |  |  |  |
| Non-Hispanic White | 1.0430 | 2.3293 | 3.8758 | 2.1285 | 0.0906 | 1.8113 | 0.0880 | 1.7857 | 0.2924 | 2.8918 | 0.7799 | 1.9919 | 0.1171 | 2.0375 |  |  |  |
| Hispanic | 1.3004 | 3.6906 | 3.6223 | 3.1779 | 0.0924 | 1.8687 | 0.0852 | 1.8852 | 0.3286 | 2.9896 | 0.7373 | 2.1308 | 0.1478 | 2.2609 |  |  |  |
| Highest level of maternal education |  |  |  |  |  |  |  |  |  |  |  |  |  |  |  |  |  |
| <High School | 1.8175 | 4.1680 | 8.2367 | 2.3884 | 0.1083 | 1.8783 | 0.1214 | 1.9531 | 0.3673 | 3.2981 | 1.2533 | 1.9749 | 0.2013 | 2.1311 |  |  |  |
| High School/GED/Technical School | 1.4745 | 3.4093 | 6.0291 | 2.2610 | 0.1073 | 1.8710 | 0.1101 | 1.8122 | 0.3096 | 3.1025 | 1.1269 | 1.9135 | 0.1752 | 2.0252 |  |  |  |
| College or Higher | 1.0974 | 2.8775 | 4.2248 | 2.3145 | 0.0978 | 2.0399 | 0.0954 | 1.9848 | 0.2971 | 3.1480 | 0.8458 | 2.1279 | 0.1211 | 2.1916 |  |  |  |
| Prior births |  |  |  |  |  |  |  |  |  |  |  |  |  |  |  |  |  |
| Yes | 1.2376 | 3.2207 | 5.4164 | 2.4178 | 0.1061 | 1.9243 | 0.1050 | 1.8626 | 0.3163 | 3.1581 | 1.0494 | 2.1207 | 0.1578 | 2.1285 |  |  |  |
| No | 1.5125 | 3.3874 | 5.3124 | 2.4053 | 0.0997 | 1.9699 | 0.1048 | 1.9644 | 0.2960 | 3.1368 | 0.9717 | 1.9359 | 0.1498 | 2.1695 |  |  |  |
| Pre-pregnancy BMI |  |  |  |  |  |  |  |  |  |  |  |  |  |  |  |  |  |
| Underweight | 1.1239 | 2.0816 | 3.4857 | 2.1225 | 0.0757 | 1.6822 | 0.0872 | 1.7091 | 0.2767 | 2.5888 | 0.7973 | 1.9502 | 0.1512 | 2.0964 |  |  |  |
| Normal | 1.3047 | 3.1325 | 4.6438 | 2.2742 | 0.0930 | 1.8907 | 0.1030 | 1.9291 | 0.2813 | 3.1027 | 0.9293 | 2.0783 | 0.1310 | 2.1444 |  |  |  |
| Overweight | 1.4006 | 3.2515 | 5.8435 | 2.1889 | 0.1011 | 1.8229 | 0.1056 | 1.8418 | 0.3151 | 3.1199 | 1.0125 | 1.8975 | 0.1583 | 2.0138 |  |  |  |
| Obese | 1.3598 | 3.6579 | 6.2308 | 2.6530 | 0.1224 | 2.0426 | 0.1087 | 1.9375 | 0.3375 | 3.2839 | 1.1573 | 2.0857 | 0.1823 | 2.1668 |  |  |  |
| Maternal Childhood Traumatic Life Events |  |  |  |  |  |  |  |  |  |  |  |  |  |  |  |  |  |
| 0 | 1.3000 | 3.3732 | 5.3380 | 2.4071 | 0.1019 | 1.9063 | 0.1030 | 1.9187 | 0.2946 | 3.1797 | 0.9891 | 2.0676 | 0.1450 | 2.1644 |  |  |  |
| 1 | 1.4412 | 3.1157 | 5.4077 | 2.3565 | 0.1022 | 1.9090 | 0.1063 | 1.8220 | 0.2999 | 3.0668 | 1.0686 | 1.9665 | 0.1657 | 2.0252 |  |  |  |
| 2 or 3 | 1.4097 | 3.1372 | 5.5084 | 2.5791 | 0.1158 | 2.2276 | 0.1145 | 1.9958 | 0.4182 | 3.1415 | 1.0606 | 2.1511 | 0.1878 | 2.2301 |  |  |  |
| Fetal sex |  |  |  |  |  |  |  |  |  |  |  |  |  |  |  |  |  |
| Female | 1.3064 | 3.1171 | 5.1275 | 2.3643 | 0.1011 | 1.9005 | 0.1041 | 1.9034 | 0.2968 | 3.2372 | 1.0099 | 2.0330 | 0.1553 | 2.1846 |  |  |  |
| Male | 1.3820 | 3.4987 | 5.6514 | 2.4580 | 0.1061 | 1.9888 | 0.1058 | 1.9049 | 0.3202 | 3.0548 | 1.0250 | 2.0654 | 0.1537 | 2.1043 |  |  |  |

**Supplementary Table 3. p-values for the cross-product terms of OH-PAH metabolites by potential effect modifiers in mixed effect models examining log-transformed OH-PAH metabolite concentrations in relation to pCRH concentrations across mid-late pregnancy.**

|  | **Potential Effect Modifiers** | | | |
| --- | --- | --- | --- | --- |
| **OH-PAH metabolite^1,2^** | **Fetal sex**  **(n=797)** | **Maternal childhood traumatic events (n=797)** | **Gestational diabetes**  **(n=792)** | **Gestational hypertension (n=792)** |
| ***pCRH*** | p-value | p-value | p-value | p-value |
| 1-OH-NAP | 0.13 | 0.66 | 0.53 | 0.70 |
| 2-OH-NAP | 0.12 | 0.17 | 0.83 | 0.63 |
| 2-OH-PHEN | 0.39 | 0.60 | 0.89 | 0.94 |
| 3-OH-PHEN | 0.40 | 0.64 | 0.79 | 0.48 |
| 1/9-OH-PHEN | 0.62 | 0.62 | 0.44 | 0.32 |
| 2/3/9-OH-FLUO | 0.17 | 0.51 | 0.57 | 0.71 |
| 1-OH-PYR | 0.14 | 0.77 | 0.36 | 0.45 |
| 𝛴NAP | 0.10 | 0.41 | 0.95 | 0.59 |
| 𝛴PHEN | 0.49 | 0.77 | 0.70 | 0.39 |

1 Abbreviations: 1-OH-NAP= 1-OH-Naphthalene; 2-OH-NAP=2-OH-Naphthalene; 2-OH-PHEN= 2-OH-Phenanthrene; 3-OH-PHEN= 3-OH-Phenanthrene; 1/9-OH-PHEN=combined 1/9-OH-Phenanthrene; 2/3/9-OH-FLUO= combined 2/3/9-OH-Fluorene; 1-OH-PYR= 1-OH-Pyrene; 𝛴NAP=sum of 1-OH-NAP and 2-OH-NAP; 𝛴PHEN=sum of 2-OH-PHEN, 3-OH-PHEN, 4-OH-PHEN, and 1/9-OH-PHEN.

2 Models include gestational age at blood collection, specific gravity, maternal age, race/ethnicity, education, pre-pregnancy BMI, cotinine, fetal sex, parity, childhood traumatic events, gestational diabetes, and gestational hypertension as well as interaction terms for each of the covariates * gestational age at blood collection.

**Supplementary Table 4. Linear regression models examining log-transformed OH-PAH metabolite concentrations in relation to pCRH concentrations at individual time points (Visit 1 and Visit 2; in pg/mL).**

| **OH-PAH metabolite^1^** | **Minimally adjusted model^2^**  **β (95% CI); p-value** | **Fully adjusted model ^3^**  **β (95% CI); p-value** | **Extended model ^4^**  **β (95% CI); p-value** |
| --- | --- | --- | --- |
| ***pCRH at Visit 1*** | N=812 | N=797 | N=790 |
| 1-OH-NAP | -0.03 (-0.07, 0.01); 0.16 | -0.001 (-0.04, 0.04); 0.96 | -0.001 (-0.04, 0.04); 0.98 |
| 2-OH-NAP | -0.04 (-0.10, 0.01); 0.12 | 0.03 (-0.02, 0.09); 0.26 | 0.03 (-0.03, 0.09); 0.29 |
| 2-OH-PHEN | -0.07 (-0.14, 0.01); 0.08 | 0.01 (-0.07, 0.08); 0.90 | 0.01 (-0.06, 0.08); 0.84 |
| 3-OH-PHEN | -0.09 (-0.16, -0.01); 0.02 | -0.02 (-0.09, 0.06); 0.69 | -0.01 (-0.09, 0.06); 0.77 |
| 1/9-OH-PHEN | 0.01 (-0.03, 0.05); 0.65 | 0.02 (-0.02, 0.05); 0.44 | 0.02 (-0.02, 0.06); 0.34 |
| 2/3/9-OH-FLUO | -0.07 (-0.13, -0.001); 0.05 | 0.01 (-0.05, 0.08); 0.70 | 0.01 (-0.05, 0.08); 0.68 |
| 1-OH-PYR | -0.10 (-0.16, -0.03); 0.004 | -0.01 (-0.07, 0.05); 0.80 | -0.004 (-0.07, 0.06); 0.91 |
| 𝛴NAP | -0.06 (-0.12, -0.01); 0.03 | 0.02 (-0.03, 0.08); 0.46 | 0.02 (-0.04, 0.07); 0.50 |
| 𝛴PHEN | -0.01 (-0.08, 0.05); 0.71 | 0.02 (-0.04, 0.09); 0.49 | 0.03 (-0.04, 0.09); 0.38 |
| ***pCRH at Visit 2*** | N=802 | N=795 | N=790 |
| 1-OH-NAP | -0.03 (-0.07, 0.02); 0.26 | -0.002 (-0.05, 0.04); 0.93 | 0.001 (-0.04, 0.04); 0.98 |
| 2-OH-NAP | -0.03 (-0.09, 0.03); 0.35 | 0.05 (-0.02, 0.11); 0.17 | 0.04 (-0.02, 0.11); 0.19 |
| 2-OH-PHEN | -0.03 (-0.12, 0.05); 0.45 | 0.03 (-0.05, 0.12); 0.43 | 0.03 (-0.05, 0.12); 0.44 |
| 3-OH-PHEN | -0.06 (-0.15, 0.02); 0.15 | 0.003 (-0.08, 0.09); 0.95 | 0.01 (-0.07, 0.09); 0.82 |
| 1/9-OH-PHEN | 0.02 (-0.03, 0.07); 0.42 | 0.03 (-0.02, 0.07); 0.28 | 0.03 (-0.01, 0.08); 0.14 |
| 2/3/9-OH-FLUO | -0.05 (-0.12, 0.03); 0.20 | 0.03 (-0.05, 0.10); 0.50 | 0.02 (-0.06, 0.09); 0.67 |
| 1-OH-PYR | -0.08 (-0.15, -0.004); 0.04 | 0.005 (-0.07, 0.08); 0.90 | 0.03 (-0.03, 0.10); 0.30 |
| 𝛴NAP | -0.05 (-0.11, 0.02); 0.16 | 0.03 (-0.03, 0.10); 0.30 | 0.03 (-0.03, 0.10); 0.30 |
| 𝛴PHEN | -0.001 (-0.08, 0.08); 0.99 | 0.03 (-0.04, 0.11); 0.42 | 0.04 (-0.03, 0.12); 0.25 |

1 Abbreviations: 1-OH-NAP= 1-OH-Naphthalene; 2-OH-NAP=2-OH-Naphthalene; 2-OH-PHEN= 2-OH-Phenanthrene; 3-OH-PHEN= 3-OH-Phenanthrene; 1/9-OH-PHEN=combined 1/9-OH-Phenanthrene; 2/3/9-OH-FLUO= combined 2/3/9-OH-Fluorene; 1-OH-PYR= 1-OH-Pyrene; 𝛴NAP=sum of 1-OH-NAP and 2-OH-NAP; 𝛴PHEN=sum of 2-OH-PHEN, 3-OH-PHEN, 4-OH-PHEN, and 1/9-OH-PHEN.

2 Minimally adjusted models include gestational age at blood collection, specific gravity

3 Fully adjusted models include gestational age at blood collection, specific gravity, maternal age, race/ethnicity, education, pre-pregnancy BMI, cotinine, fetal sex, parity, and childhood traumatic events.

4 Extended models include gestational age at blood collection, specific gravity, maternal age, race/ethnicity, education, pre-pregnancy BMI, cotinine, fetal sex, parity, childhood traumatic events, gestational diabetes, and gestational hypertension.

**Supplementary Table 5. Mixed effect models examining log-transformed PAH metabolite concentrations in relation to pCRH concentrations across mid-late pregnancy including smokers and participants with** **urinary cotinine >200 ng/mL.**

| **OH-PAH metabolite^1,2^** | **Minimally adjusted model^3^**  **β (95% CI); p-value** | **Fully adjusted model ^4^**  **β (95% CI); p-value** | **Extended model ^5^**  **β (95% CI); p-value** |
| --- | --- | --- | --- |
| ***pCRH*** | N=930 | N=911 | N=906 |
| 1-OH-NAP | 0.0003 (-0.0001, 0.0007); 0.10 | 0.0002 (-0.0002, 0.0006); 0.36 | 0.0003 (-0.0001, 0.0006); 0.20 |
| 2-OH-NAP | 0.0005 (0.00001, 0.0010); 0.04 | 0.0004 (-0.0002, 0.0009); 0.20 | 0.0004 (-0.0002, 0.0010); 0.17 |
| 2-OH-PHEN | 0.0011 (0.0004, 0.0018); 0.002 | 0.0009 (0.0002, 0.0017); 0.01 | 0.0009 (0.0002, 0.0016); 0.01 |
| 3-OH-PHEN | 0.0010 (0.0003, 0.0017); 0.005 | 0.0007 (0.0001, 0.0015); 0.05 | 0.0008(-0.0001, 0.0015); 0.03 |
| 1/9-OH-PHEN | 0.0002 (-0.0002, 0.0006); 0.43 | 0.0001 (-0.0003, 0.0005); 0.49 | 0.0002 (-0.0002, 0.0006); 0.29 |
| 2/3/9-OH-FLUO | 0.0006 (0.0001, 0.0011); 0.03 | 0.0003 (-0.0003, 0.0010); 0.34 | 0.0003 (-0.0003, 0.0009); 0.34 |
| 1-OH-PYR | 0.0007 (0.0001, 0.0013); 0.01 | 0.0005 (-0.0002, 0.0011); 0.14 | 0.0006 (0.00004, 0.0012); 0.06 |
| 𝛴NAP | 0.0006 (0.0001, 0.0011); 0.19 | 0.0005 (-0.0001, 0.0010); 0.10 | 0.0005 (-0.00003, 0.0011); 0.06 |
| 𝛴PHEN | 0.0005 (-0.0001, 0.0012); 0.11 | 0.0004 (-0.0003, 0.0011); 0.26 | 0.0005 (-0.0002, 0.0012); 0.14 |

1 Abbreviations: 1-OH-NAP= 1-OH-Naphthalene; 2-OH-NAP=2-OH-Naphthalene; 2-OH-PHEN= 2-OH-Phenanthrene; 3-OH-PHEN= 3-OH-Phenanthrene; 1/9-OH-PHEN=combined 1/9-OH-Phenanthrene; 2/3/9-OH-FLUO= combined 2/3/9-OH-Fluorene; 1-OH-PYR= 1-OH-Pyrene; 𝛴NAP=sum of 1-OH-NAP and 2-OH-NAP; 𝛴PHEN=sum of 2-OH-PHEN, 3-OH-PHEN, 4-OH-PHEN, and 1/9-OH-PHEN.

2 Coefficients, 95% confidence intervals and the p-values for the interaction between each of the log-transformed PAHs and time (repeated measures of gestational age at blood collection) are reported.

3 Minimally adjusted models include gestational age at blood collection, specific gravity

4 Fully adjusted models include gestational age at blood collection, specific gravity, maternal age, race/ethnicity, education, pre-pregnancy BMI, cotinine, fetal sex, parity, and childhood traumatic events as well as interaction terms for each of the covariates * gestational age at blood collection.

5 Extended models include gestational age at blood collection, specific gravity, maternal age, race/ethnicity, education, pre-pregnancy BMI, cotinine, fetal sex, parity, childhood traumatic events, gestational diabetes, and gestational hypertension as well as interaction terms for each of the covariates * gestational age at blood collection.

**Supplementary Table 6. Mixed effect models examining log-transformed PAH metabolite concentrations in relation to pCRH concentrations across mid-late pregnancy restricted to participants who delivered at term (>37 weeks and <42 weeks).**

| **OH-PAH metabolite^1,2^** | **Minimally adjusted model^3^**  **β (95% CI); p-value** | **Fully adjusted model ^4^**  **β (95% CI); p-value** | **Extended model ^5^**  **β (95% CI); p-value** |
| --- | --- | --- | --- |
| ***pCRH*** | N=744 | N=734 | N=731 |
| 1-OH-NAP | 0.0003 (-0.0002, 0.0007); 0.24 | 0.0002 (-0.0002, 0.0007); 0.33 | 0.0003 (-0.0002, 0.0007); 0.23 |
| 2-OH-NAP | 0.0003 (-0.0003, 0.0009); 0.29 | 0.0002 (-0.0004, 0.0009); 0.44 | 0.0002 (-0.0004, 0.0009); 0.42 |
| 2-OH-PHEN | 0.0011 (0.0003, 0.0019); 0.01 | 0.0010 (0.0001, 0.0018); 0.02 | 0.0008 (0.00001, 0.0016); 0.05 |
| 3-OH-PHEN | 0.0010 (0.0001, 0.0018); 0.03 | 0.0008 (-0.0001, 0.0016); 0.07 | 0.0007(-0.0001, 0.0015); 0.10 |
| 1/9-OH-PHEN | 0.0002 (-0.0002, 0.0007); 0.32 | 0.0003 (-0.0002, 0.0007); 0.23 | 0.0003 (-0.0001, 0.0007); 0.17 |
| 2/3/9-OH-FLUO | 0.0004 (-0.0004, 0.0011); 0.33 | 0.0001 (-0.0006, 0.0009); 0.77 | 0.0001 (-0.0006, 0.0008); 0.81 |
| 1-OH-PYR | 0.0009 (0.0002, 0.0016); 0.01 | 0.0007 (-0.0001, 0.0014); 0.07 | 0.0007 (0.0000, 0.0014); 0.06 |
| 𝛴NAP | 0.0005 (-0.0001, 0.0010); 0.12 | 0.0004 (-0.0002, 0.0010); 0.20 | 0.0004 (-0.0002, 0.0010); 0.16 |
| 𝛴PHEN | 0.0005 (-0.0002, 0.0013); 0.18 | 0.0005 (-0.0003, 0.0012); 0.21 | 0.0005 (-0.0003, 0.0012); 0.21 |

1 Abbreviations: 1-OH-NAP= 1-OH-Naphthalene; 2-OH-NAP=2-OH-Naphthalene; 2-OH-PHEN= 2-OH-Phenanthrene; 3-OH-PHEN= 3-OH-Phenanthrene; 1/9-OH-PHEN=combined 1/9-OH-Phenanthrene; 2/3/9-OH-FLUO= combined 2/3/9-OH-Fluorene; 1-OH-PYR= 1-OH-Pyrene; 𝛴NAP=sum of 1-OH-NAP and 2-OH-NAP; 𝛴PHEN=sum of 2-OH-PHEN, 3-OH-PHEN, 4-OH-PHEN, and 1/9-OH-PHEN.

2 Coefficients, 95% confidence intervals and the p-values for the interaction between each of the log-transformed PAHs and time (repeated measures of gestational age at blood collection) are reported.

3 Minimally adjusted models include gestational age at blood collection, specific gravity

4 Fully adjusted models include gestational age at blood collection, specific gravity, maternal age, race/ethnicity, education, pre-pregnancy BMI, cotinine, fetal sex, parity, and childhood traumatic events as well as interaction terms for each of the covariates * gestational age at blood collection.

5 Extended models include gestational age at blood collection, specific gravity, maternal age, race/ethnicity, education, pre-pregnancy BMI, cotinine, fetal sex, parity, childhood traumatic events, gestational diabetes, and gestational hypertension as well as interaction terms for each of the covariates * gestational age at blood collection.

**Supplementary Table 7. Mixed effect models examining log-transformed PAH metabolite concentrations in relation to pCRH concentrations across mid-late pregnancy restricted to participants with no history of preterm birth.**

| **OH-PAH metabolite^1,2^** | **Minimally adjusted model^3^**  **β (95% CI); p-value** | **Fully adjusted model ^4^**  **β (95% CI); p-value** | **Extended model ^5^**  **β (95% CI); p-value** |
| --- | --- | --- | --- |
| ***pCRH*** | N=752 | N=740 | N=736 |
| 1-OH-NAP | 0.0002 (-0.0002, 0.0007); 0.27 | 0.0002 (-0.0002, 0.0006); 0.34 | 0.0003 (-0.0002, 0.0007); 0.23 |
| 2-OH-NAP | 0.0003 (-0.0003, 0.0009); 0.32 | 0.0003 (-0.0003, 0.0009); 0.37 | 0.0003 (-0.0003, 0.0009); 0.31 |
| 2-OH-PHEN | 0.0010 (0.0002, 0.0018); 0.02 | 0.0009 (0.0001, 0.0018); 0.02 | 0.0009 (0.0001, 0.0017); 0.03 |
| 3-OH-PHEN | 0.0008 (-0.000002, 0.0017); 0.05 | 0.0008 (-0.0001, 0.0016); 0.08 | 0.0008(-0.00004, 0.0016); 0.06 |
| 1/9-OH-PHEN | 0.0001 (-0.0003, 0.0006); 0.53 | 0.0002 (-0.0003, 0.0006); 0.44 | 0.0002 (-0.0002, 0.0007); 0.31 |
| 2/3/9-OH-FLUO | 0.0002 (-0.0005, 0.0009); 0.56 | 0.0001 (-0.0007, 0.0008); 0.82 | 0.0001 (-0.0006, 0.0009); 0.72 |
| 1-OH-PYR | 0.0008 (0.0001, 0.0015); 0.02 | 0.0008 (0.0001, 0.0015); 0.03 | 0.0008 (0.0001, 0.0015); 0.02 |
| 𝛴NAP | 0.0004 (-0.0001, 0.0010); 0.13 | 0.0004 (-0.0002, 0.0010); 0.18 | 0.0005 (-0.0001, 0.0011); 0.11 |
| 𝛴PHEN | 0.0004 (-0.0004, 0.0011); 0.34 | 0.0004 (-0.0004, 0.0011); 0.35 | 0.0004 (-0.0003, 0.0012); 0.26 |

1 Abbreviations: 1-OH-NAP= 1-OH-Naphthalene; 2-OH-NAP=2-OH-Naphthalene; 2-OH-PHEN= 2-OH-Phenanthrene; 3-OH-PHEN= 3-OH-Phenanthrene; 1/9-OH-PHEN=combined 1/9-OH-Phenanthrene; 2/3/9-OH-FLUO= combined 2/3/9-OH-Fluorene; 1-OH-PYR= 1-OH-Pyrene; 𝛴NAP=sum of 1-OH-NAP and 2-OH-NAP; 𝛴PHEN=sum of 2-OH-PHEN, 3-OH-PHEN, 4-OH-PHEN, and 1/9-OH-PHEN.

2 Coefficients, 95% confidence intervals and the p-values for the interaction between each of the log-transformed PAHs and time (repeated measures of gestational age at blood collection) are reported.

3 Minimally adjusted models include gestational age at blood collection, specific gravity

4 Fully adjusted models include gestational age at blood collection, specific gravity, maternal age, race/ethnicity, education, pre-pregnancy BMI, cotinine, fetal sex, parity, and childhood traumatic events as well as interaction terms for each of the covariates * gestational age at blood collection.

5 Extended models include gestational age at blood collection, specific gravity, maternal age, race/ethnicity, education, pre-pregnancy BMI, cotinine, fetal sex, parity, childhood traumatic events, gestational diabetes, and gestational hypertension as well as interaction terms for each of the covariates * gestational age at blood collection.

**Supplementary Table 8. Mixed effect models examining log-transformed PAH metabolite concentrations in relation to pCRH concentrations across mid-late pregnancy without adjustment for maternal childhood traumatic events.**

| **OH-PAH metabolite^1,2^** | **Minimally adjusted model^3^**  **β (95% CI); p-value** | **Fully adjusted model ^4^**  **β (95% CI); p-value** | **Extended model ^5^**  **β (95% CI); p-value** |
| --- | --- | --- | --- |
| ***pCRH*** | N=812 | N=805 | N=800 |
| 1-OH-NAP | 0.0002 (-0.0002, 0.0006); 0.34 | 0.0002 (-0.0003, 0.0006); 0.43 | 0.0003 (-0.0002, 0.0007); 0.23 |
| 2-OH-NAP | 0.0003 (-0.0002, 0.0009); 0.25 | 0.0003 (-0.0004, 0.0009); 0.41 | 0.0003 (-0.0003, 0.0009); 0.39 |
| 2-OH-PHEN | 0.0010 (0.0002, 0.0018); 0.01 | 0.0009 (0.0001, 0.0017); 0.03 | 0.0008 (0.00002, 0.0016); 0.04 |
| 3-OH-PHEN | 0.0008 (-0.00002, 0.0016); 0.06 | 0.0007 (-0.0001, 0.0015); 0.11 | 0.0007(-0.0001, 0.0016); 0.07 |
| 1/9-OH-PHEN | 0.0002 (-0.0003, 0.0006); 0.47 | 0.0002 (-0.0002, 0.0006); 0.32 | 0.0003 (-0.0001, 0.0007); 0.15 |
| 2/3/9-OH-FLUO | 0.0003 (-0.0004, 0.0010); 0.47 | 0.0001 (-0.0006, 0.0008); 0.79 | 0.0001 (-0.0006, 0.0008); 0.77 |
| 1-OH-PYR | 0.0007 (-0.000003, 0.0013); 0.05 | 0.0006 (0.0001, 0.0013); 0.12 | 0.0007 (-0.00001, 0.0014); 0.05 |
| 𝛴NAP | 0.0005 (-0.0001, 0.0010); 0.12 | 0.0004 (-0.0002, 0.0010); 0.22 | 0.0004 (-0.0002, 0.0010); 0.15 |
| 𝛴PHEN | 0.0004 (-0.0003, 0.0011); 0.31 | 0.0004 (-0.0003, 0.0011); 0.30 | 0.0005 (-0.0002, 0.0012); 0.16 |

1 Abbreviations: 1-OH-NAP= 1-OH-Naphthalene; 2-OH-NAP=2-OH-Naphthalene; 2-OH-PHEN= 2-OH-Phenanthrene; 3-OH-PHEN= 3-OH-Phenanthrene; 1/9-OH-PHEN=combined 1/9-OH-Phenanthrene; 2/3/9-OH-FLUO= combined 2/3/9-OH-Fluorene; 1-OH-PYR= 1-OH-Pyrene; 𝛴NAP=sum of 1-OH-NAP and 2-OH-NAP; 𝛴PHEN=sum of 2-OH-PHEN, 3-OH-PHEN, 4-OH-PHEN, and 1/9-OH-PHEN.

2 Coefficients, 95% confidence intervals and the p-values for the interaction between each of the log-transformed PAHs and time (repeated measures of gestational age at blood collection) are reported.

3 Minimally adjusted models include gestational age at blood collection, specific gravity

4 Fully adjusted models include gestational age at blood collection, specific gravity, maternal age, race/ethnicity, education, pre-pregnancy BMI, cotinine, fetal sex, and parity, as well as interaction terms for each of the covariates * gestational age at blood collection.

5 Extended models include gestational age at blood collection, specific gravity, maternal age, race/ethnicity, education, pre-pregnancy BMI, cotinine, fetal sex, parity, gestational diabetes, and gestational hypertension as well as interaction terms for each of the covariates * gestational age at blood collection.

**Supplementary Table 9. Mixed effect models examining log-transformed PAH metabolite concentrations in relation to pCRH concentrations across mid-late pregnancy restricted to participants with Visit 1 before gestational age 27 weeks and Visit 2 after gestational age 27 weeks.**

| **OH-PAH metabolite^1,2^** | **Minimally adjusted model^3^**  **β (95% CI); p-value** | **Fully adjusted model ^4^**  **β (95% CI); p-value** | **Extended model ^5^**  **β (95% CI); p-value** |
| --- | --- | --- | --- |
| ***pCRH*** | N=713 | N=707 | N=702 |
| 1-OH-NAP | 0.0002 (-0.0002, 0.0006); 0.34 | 0.0002 (-0.0003, 0.0006); 0.47 | 0.0003 (-0.0002, 0.0007); 0.25 |
| 2-OH-NAP | 0.0002 (-0.0004, 0.0008); 0.41 | 0.0002 (-0.0004, 0.0008); 0.52 | 0.0002 (-0.0004, 0.0008); 0.50 |
| 2-OH-PHEN | 0.0009 (0.0001, 0.0017); 0.02 | 0.0008 (-0.0003, 0.0016); 0.06 | 0.0007 (-0.0001, 0.0016); 0.07 |
| 3-OH-PHEN | 0.0008 (-0.00006, 0.0016); 0.07 | 0.0006 (-0.0002, 0.0015); 0.13 | 0.0007(-0.0001, 0.0016); 0.09 |
| 1/9-OH-PHEN | 0.0001 (-0.0003, 0.0006); 0.57 | 0.0002 (-0.0003, 0.0006); 0.47 | 0.0003 (-0.0001, 0.0007); 0.22 |
| 2/3/9-OH-FLUO | 0.0003 (-0.0004, 0.0010); 0.43 | 0.0001 (-0.0006, 0.0009); 0.71 | 0.0001 (-0.0006, 0.0009); 0.69 |
| 1-OH-PYR | 0.0006 (-0.0001, 0.0013); 0.10 | 0.0004 (-0.0003, 0.0012); 0.24 | 0.0006 (-0.0002, 0.0013); 0.12 |
| 𝛴NAP | 0.0004 (-0.0002, 0.0010); 0.21 | 0.0003 (-0.0003, 0.0009); 0.30 | 0.0004 (-0.0002, 0.0010); 0.21 |
| 𝛴PHEN | 0.0003 (-0.0004, 0.0011); 0.40 | 0.0003 (-0.0005, 0.0010); 0.47 | 0.0004 (-0.0003, 0.0012); 0.26 |

1 Abbreviations: 1-OH-NAP= 1-OH-Naphthalene; 2-OH-NAP=2-OH-Naphthalene; 2-OH-PHEN= 2-OH-Phenanthrene; 3-OH-PHEN= 3-OH-Phenanthrene; 1/9-OH-PHEN=combined 1/9-OH-Phenanthrene; 2/3/9-OH-FLUO= combined 2/3/9-OH-Fluorene; 1-OH-PYR= 1-OH-Pyrene; 𝛴NAP=sum of 1-OH-NAP and 2-OH-NAP; 𝛴PHEN=sum of 2-OH-PHEN, 3-OH-PHEN, 4-OH-PHEN, and 1/9-OH-PHEN.

2 Coefficients, 95% confidence intervals and the p-values for the interaction between each of the log-transformed PAHs and time (repeated measures of gestational age at blood collection) are reported.

3 Minimally adjusted models include gestational age at blood collection, specific gravity

4 Fully adjusted models include gestational age at blood collection, specific gravity, maternal age, race/ethnicity, education, pre-pregnancy BMI, cotinine, fetal sex, parity, and childhood traumatic events as well as interaction terms for each of the covariates * gestational age at blood collection.

5 Extended models include gestational age at blood collection, specific gravity, maternal age, race/ethnicity, education, pre-pregnancy BMI, cotinine, fetal sex, parity, childhood traumatic events, gestational diabetes, and gestational hypertension as well as interaction terms for each of the covariates * gestational age at blood collection.
